# A Phytochemical-Sensing Strategy Based on Mass Spectrometry Imaging and Metabolic Profiling for Understanding the Functionality of the Medicinal Herb Green Tea

**DOI:** 10.3390/molecules22101621

**Published:** 2017-09-27

**Authors:** Yoshinori Fujimura, Daisuke Miura, Hirofumi Tachibana

**Affiliations:** Division of Applied Biological Chemistry, Department of Bioscience and Biotechnology, Faculty of Agriculture, Kyushu University, 6-10-1 Hakozaki, Higashi-ku, Fukuoka 812-8581, Japan; tatibana@agr.kyushu-u.ac.jp

**Keywords:** mass spectrometry imaging, metabolic profiling, phytochemical, metabolite, medicinal herb, green tea, catechin, food functionality

## Abstract

Low-molecular-weight phytochemicals have health benefits and reduce the risk of diseases, but the mechanisms underlying their activities have remained elusive because of the lack of a methodology that can easily visualize the exact behavior of such small molecules. Recently, we developed an in situ label-free imaging technique, called mass spectrometry imaging, for visualizing spatially-resolved biotransformations based on simultaneous mapping of the major bioactive green tea polyphenol and its phase II metabolites. In addition, we established a mass spectrometry-based metabolic profiling technique capable of evaluating the bioactivities of diverse green tea extracts, which contain multiple phytochemicals, by focusing on their compositional balances. This methodology allowed us to simultaneously evaluate the relative contributions of the multiple compounds present in a multicomponent system to its bioactivity. This review highlights small molecule-sensing techniques for visualizing the complex behaviors of herbal components and linking such information to an enhanced understanding of the functionalities of multicomponent medicinal herbs.

## 1. Introduction

Many functionality-oriented botanicals, nutraceuticals, and foods have been developed for widespread consumption, although some have been included in health hazard evaluation reports on the inadequate and excessive consumption of a specific ingredient with functionality [1,2]. At present, there are few reliable quantitative evaluation methods for predicting the efficacy, safety, and dosing of dietary components. Detailed knowledge about the exact composition of a food product or medicinal herb, the active or inactive ingredient(s), and their metabolism and mechanism of action is frequently lacking [3]. This issue has arisen in part because of limitations with the analytical techniques available for detecting complex behaviors of dietary factors in the body, foods, and medicinal herbs. To begin to overcome this, methods with improved detection sensitivity, accuracy, specificity, coverage, and spatiotemporal resolution are required. Elucidation of specific cellular signaling pathways mediated through a food factor- or phytochemical-sensing molecule and the spatiotemporal dynamics of a dietary factor and its metabolites in the tissue micro-regions will enhance understanding of the underlying mechanism of action and exact pharmacokinetics (or nutrikinetics) [4,5,6]. Pharmacology of botanical-based multicomponent nutraceuticals requires a “network” approach, in which multiple compounds interact with multiple targets in the body with interdependent activities to achieve an optimal effect. The traditional approach to understanding the pharmacology of a multicomponent nutraceutical involves studying the effects of a single component on a single biological activity, and gradually assembling those effects into an integrated picture. However, assembling the results obtained from such a reductionistic approach to achieve a system-wide understanding of a concerted pharmacological intervention has proven impractical [7]. Other methods capable of simultaneously evaluating the relative contributions of multiple compounds in a nutraceutical to its pharmacological activity are needed. Techniques capable of directly attributing the behavior of both single components and multiple components in food products or medicinal herbs to physiological function will be indispensable for clarifying the exact functions of botanicals, nutraceuticals, and foods in multicomponent systems [8].

Tea is one of the most widely consumed beverages in the world after water. Green tea, black tea, and oolong tea are all derived from the dried leaves of the plant *Camellia sinensis*. Among the teas, green tea has been studied the most for its health benefits. Green tea (*Camellia sinensis* L.) is an important medicinal herb containing low-molecular-weight compounds called phytochemicals. Over the past two decades, it has emerged as an important agricultural product and source of dietary factor for health maintenance and promotion, disease risk reduction, and chemotherapy [9]. Because of their beneficial health effects, the intake of tea infusion and its phytochemicals is considered to be an inexpensive, readily applicable, acceptable, and accessible approach to disease control and management [10]. Tea constituents exhibit various biological and pharmacological properties, such as anti-carcinogenic, anti-oxidative, anti-allergic, anti-viral, anti-hypertensive, anti-atherosclerotic, anti-cardiovascular disease, and anti-hypercholesterolemic activities [11,12,13,14,15,16,17,18]. The principle components responsible for the activities of green tea extract (GTE) are catechins, which are polyphenols. The most abundant and bioactive green tea catechin is (−)-epigallocatechin-3-*O*-gallate (EGCG).

In the field of biomedical and food sciences, both chemical biology and metabolomics methods are gradually becoming recognized as powerful approaches for precise and high-resolution analysis of small molecule behavior. This review describes two new small molecule behavior-sensing techniques suitable for analysis in cells, tissues, foods, and medicinal herbs. These techniques can be used to increase understanding of the functionality of the GTE multiple component system and its major small molecule components such as EGCG.

## 2. In Situ Label-Free Imaging of Orally Administered Phytochemicals

### 2.1. Mass Spectrometry Imaging

**Figure 1:**
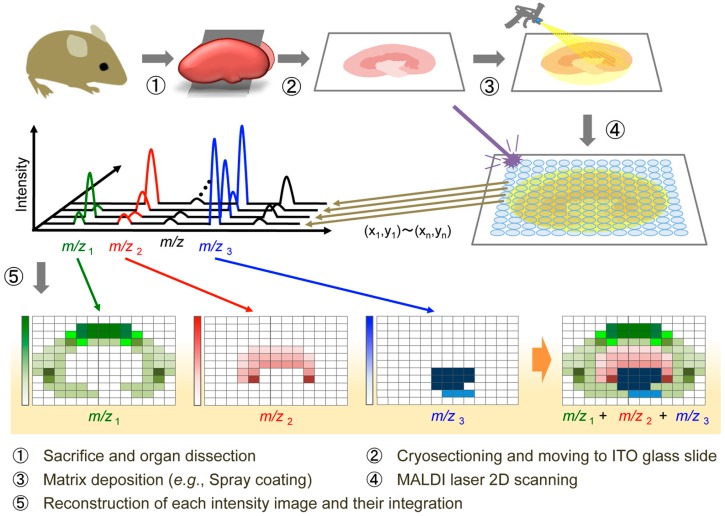
Schematic representation of the MALDI-MSI experimental procedure.

To elucidate the precise mechanism underlying phytochemical bioactivity, high-resolution spatiotemporal information is needed. Although some studies have visualized the tissue distributions of phytochemicals by fluorescence imaging, cerium chloride staining, immunostaining, and radioactive labeling assays [19,20,21,22], spatiotemporal information is lacking because of the absence of an analytical technique that can easily detect the localization of the naïve polyphenol. Conventional molecular imaging generally requires labeling steps that are time-consuming, expensive, and labor-intensive. Additionally, the molecular discriminating powers of these techniques are insufficient for visualizing the target compound and its metabolites simultaneously. A label-free molecular imaging technique could overcome these issues, but the development of such a technique has been challenging. Mass spectrometry imaging (MSI) is a new technology capable of determining the naïve distribution of ionizable biological molecules in tissue sections based on their specific mass-to-charge ratios without any labeling. This technique can theoretically detect target molecules and their metabolites simultaneously in a single analysis, and is now widely used for in situ imaging of endogenous and exogenous molecules such as proteins, lipids, drugs, and their metabolites [23,24,25]. It shows potential tool useful for pathological analysis and the understanding of diseases or pharmaceutical mechanisms of action. Matrix-assisted laser desorption/ionization (MALDI), a commonly available ionization method used for MSI, is a laser desorption ionization (LDI) method that softly ionizes several biological molecules. The workflow for MALDI-MSI includes tissue preparation, matrix application, MSI data acquisition, data analysis, and image construction (Figure 1). This ionization technique is usually combined with time-of-flight (TOF)-MS. A conventional MALDI source is equipped with an ultraviolet (UV) laser, such as a nitrogen laser (337 nm) or Nd-YAG laser (355 nm). MALDI-MSI is typically performed at spatial resolutions of 10–200 μm in single organs. The spatial resolution is primarily dependent on the diameter of the laser irradiated area, which is usually more than 5 µm [26]. However, because MALDI-MSI requires a matrix application step, diffusion of metabolites within the tissue during matrix application and the heterogeneous size of crystal formation may also limit the spatial resolution.

Generally, matrix application is performed by spray coating [27,28,29] or droplet printing deposition [30,31]. Spray deposition is typically faster and offers higher spatial resolution, but the amount of solvent must be carefully controlled to prevent the tissue becoming overly wet. The droplet deposition method sacrifices resolution, which is typically no better than 200 μm because of the size of the matrix droplets. However, in this droplet deposition method, sensitivity is high because of the high analyte extraction efficiency of the droplets and there is no risk of analyte delocalization outside of the matrix spot. When applying the matrix dissolved in solvent, it is critical that the matrix spray is wet enough to extract the analytes from the tissue and into the surface matrix crystals, but not so wet that the analytes will delocalize from their original positions to neighboring regions, leading to a loss of image spatial integrity. By contrast, dry matrix application methods have been reported for imaging small molecules in tissues, and these methods minimize potential delocalization [32,33]. Vapor-phase deposition of the matrix through sublimation produced a homogeneous coating of matrix across the tissue section [34,35,36]. These experiments showed that the lipid signal was greatly enhanced, the laser spot-to-spot variation of secondary ion yield was reduced, and alkali metal contamination decreased [37]. Sublimation has the desired effect of removing any nonvolatile impurities from the matrix during the coating process [34]. However, this method has poor sensitivity because of a lack of incorporation of the analyte into the matrix [34]. To overcome this issue, Spengler et al. separated the matrix preparation procedure into two independent steps, leading to an improved sensitivity and spatial resolution [38]. The first step involved dry vapor deposition of the matrix onto the sample. In the second step, incorporation of an analyte into the matrix crystal was enhanced by controlled recrystallization of the matrix in a saturated water atmosphere. This approach achieved an effective analytical resolution of 2 μm for scanning microprobe MALDI-MS. Recent work has also demonstrated the utility of ionic liquid matrices for MALDI-MSI [39,40]. These matrices are advantageous in that there are no crystals to limit the spatial resolution.

During its first decade of use, MALDI-MS was used for synthetic polymer or protein (peptide) analysis. MALDI-MS is a highly sensitive analytical method that can be used to analyze low concentrations (approximately femtomolar) of tryptic peptides. Sensitivity is an extremely important parameter for MSI because many biological molecules are present only in very small quantities in thin tissue section. However, MALDI-MS has rarely been used for low-molecular-weight metabolite analysis because many matrix and/or matrix–analyte cluster ion peaks are observed in the low-mass range (*m*/*z* < 700), which interfere with the detection of the target compounds. Nevertheless, low-molecular-weight metabolites with distinct distributions in various tissues have been searched from large numbers of background peaks generated by conventional matrices such as 2,5-dihydroxy-benzoic acid (DHB) and α-cyano-4-hydroxycinnamic acid (CHCA). Representative low-molecular-weight metabolites and phytochemicals from edible plant tissues are shown in Table 1. γ-Aminobutyric acid (GABA) (*m*/*z* 104 [M + H]^+^) [41], α-tocopherol (*m*/*z* 431 [M + H]^+^) [42], and several anthocyanins [43,44] have been successfully detected, and their unique distributions on the surfaces of the plant tissue sections have been visualized. However, the low ionization efficiencies and interference of matrix peaks from the use of conventional matrices have made it difficult to detect other metabolites. Recently, 9-aminoacridine (9-AA) was reported as a suitable matrix for low-molecular-weight metabolite analysis [45]. When 9-AA was used in negative ionization mode, only a few peaks derived from the matrix were observed in the low-mass range (*m*/*z* ~ 500). In addition, the excellent ionization efficiency of 9-AA for important cellular metabolites (in the order of attomoles) was demonstrated [46,47]. Shroff et al. succeeded in visualizing the distribution of antiherbivore glucosinolates in *Arabidopsis thaliana* leaves using 9-AA [48]. Their results indicated that there were differences in the proportions of the three major glucosinolates in different parts of the leaf, and that their distributions appeared to control the feeding preferences of *Helicoverpa armigera* larvae. Recently, Nakamura et al. visualized the spatial distributions of metabolites within tissue sections of tomato (*Solanum lycopersicum* L.) fruit using 9-AA-MALDI-MSI combined with a matrix sublimation/recrystallization method [49]. Although apparent differences in the localization and intensity of many detected metabolites were not observed between mature green and maturered tomato fruits, the amounts of glutamate and adenosine monophosphate, which are umami compounds, increased in both the mesocarp and locule during the ripening process. By contrast, the amount of malate, a sour compound, decreased in both these regions. DHB-MALDI-MSI visualized more local metabolic alteration after wounding stress. Accumulation of a glycoalkaloid, tomatine (*m*/*z* 1034 [M + H]^+^), and a low level of its glycosylated metabolite, esculeoside A (*m*/*z* 1270 [M + H]^+^), were found in the wound region where cell death occurred. The opposite was observed in intact regions. Furthermore, the amounts of both compounds differed in developmental stages. These observations provided new insight into the physiological changes and responses of tomato fruit. MALDI-MSI using 2′,4′,6′-trihydroxyacetophenone (THAP) as the matrix has been used to investigate the spatial distribution of a class of flavonoids, (epi)catechin dimer (*m*/*z* 577 [M − H]^−^), during fruit development in strawberries (*Fragaria × ananassa*) [50]. In most cases, fruit development led to a reduction in the amounts of the investigated flavonoids in the fruit tissue, and as a consequence, they were exclusively present in the skin of mature red fruits. The natural abundance of endogenous polyphenols in high concentrations in plants, together with other UV-absorbing metabolites, allows for matrix-free UV-LDI approaches to be employed. In these cases, the plants’ own metabolites act as the endogenous MALDI matrix [51]. Hamm et al. detected resveratrol and linked stilbene compounds at the surface of grapevine leaves by LDI-TOF-MS without matrix deposition [52]. A mass-microscopic atmospheric pressure ion-source chamber for LDI imaging of freshly cut ginger rhizome sections revealed that 6-gingerol (*m*/*z* 333 [M + K]^+^) and the monoterpene (*m*/*z* 191 [M + K]^+^), which are related to pungency and flavor, were localized in oil drop-containing organelles [26].

### 2.2. Visualization of Orally Administered Phytochemicals within Mammalian Tissues

MSI is a rapidly emerging technology for visualizing the localization of exogenous drugs and their metabolites in biological tissues [58]. Compared with traditional imaging and analytical methods used in pharmaceutical research, it has several attractive advantages, especially the fact that it is completely label free. However, careful consideration is essential to select an appropriate methodology because drugs and metabolites are often more difficult to analyze in biological tissues than endogenous species because of their relatively low abundances. An important early phase of drug discovery is determining how a candidate drug is distributed and metabolized within the body. Conventionally, spatial information on the distributions of compounds in whole animals is obtained by whole-body autoradiography (typical spatial resolution 100 μm) and microautoradiography for more detailed imaging of the distributions within smaller tissues (typical spatial resolution 10 μm) [59]. In these methods, the pharmaceutical compound is labeled with a radioactive nuclide replacing a nonradioactive one brfore dosing, and this radiolabel is visualized in the tissue section. This method is highly sensitive and fully quantitative but has several disadvantages. The parent drug cannot be distinguished from its metabolites even if the metabolite contains the radiolabel. The synthesis of the drug with the incorporated label is an expensive and often time-consuming process. Furthermore, it can take several days to several weeks of exposure time to develop radiographic images of sufficient sensitivity for distribution studies. By contrast, positron-emission tomography (PET) is an in vivo imaging technique for dosed pharmaceuticals that involves radiolabeling of the drug before administration. The advantages of PET are true in vivo imaging and the ability to follow the drug distribution in real time. However, specificity is again an issue as any metabolites cannot be distinguished from the parent drug. In addition, PET suffers from relatively poor spatial resolution (approximately 1 mm for small animal studies). However, it does have the substantial advantage of being fully quantitative [60]. Compared with whole-body autoradiography, MSI can provide information on the specific localization of the analyte of interest. Thus, MSI allows for detailed localization of the parent compound and its metabolites in a single experiment without any labeling. It also offers the unique ability to co-localize drug distribution signals with endogenous analytes of interest as biological markers of disease progression, therapeutic effect, or toxicology.

Natural products derived from medicinal plants are an abundant source of biologically active phytochemicals, many of which have been used in development of pharmaceuticals and nutraceuticals [61]. To date, imaging of small molecules, including different classes of primary and secondary metabolites, is the most frequent application of plant-targeted MSI [55,62,63]. These studies will encourage an increased understanding of diverse plant biological systems and increase applications in breeding, crop improvement, and functional food or botanical drug design [42,43,44,62,64,65]. Differential distribution patterns have been evaluated for a number of molecular species, namely, lipids, amino acids, and sugars, as well as highly abundant secondary metabolites, such as polyphenols, anthocyanins, alkaloids, and glucosinolates from a variety of plant species [64]. The representative molecules from edible plants are listed in Table 1. However, there is little information on the use of MSI to follow in vivo administration of these and other bioactive dietary phytochemicals to animals. In most cases, the aforementioned dietary compounds and drugs have been detected by MALDI-MS using traditional matrices such as DHB, CHCA, and 9-AA [58,64]. These matrices are certainly effective for MALDI-MSI of limited drugs in tissue sections, but they cannot be used to easily visualize the localization of many dosed dietary compounds (food factors), including phytochemicals and their metabolites, because of their low abundance in the target tissue as well as interference with background peaks from the matrix and endogenous molecules. For effective ionization of the analyte in MALDI-MS, the optimum matrix needs to be determined because there is often no direct correlation between the choice of matrix and its ability to ionize a bioactive small molecule of interest. Kim et al. screened 41 chemicals as potential matrices for the representative bioactive green tea polyphenol, EGCG [5]. EGCG peaks were not observed with DHB, CHCA, or 9-AA. However, 1,5-diaminonaphthalene (1,5-DAN), harmane, norharmane, harmine, and ferulic acid all allowed for the detection of EGCG (*m*/*z* 457 [M − H]^−^) in negative ionization mode without any background peak interference [5]. Furthermore, among the candidate chemicals, only 1,5-DAN was useful to visualize the distribution of a single oral dose of EGCG (2000 mg/kg body weight) in mouse tissue sections. Chemical screening data that are available online could be useful for matrix selection, development of techniques for highly sensitive detection of EGCG or its derivatives, and structure-based matrix screening for MSI of dietary polyphenolic compounds.

**Figure 2:**
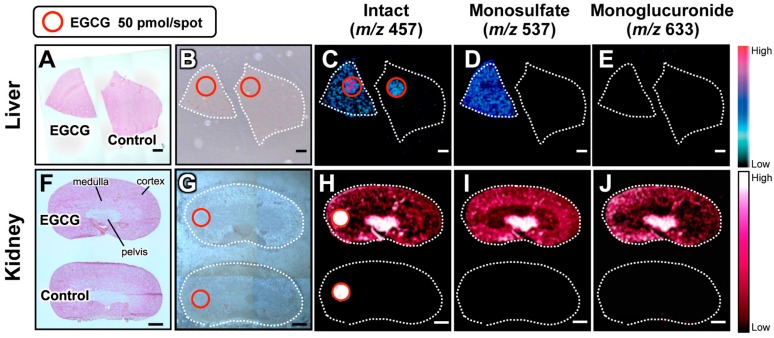
In situ label-free visualization of an orally-dosed EGCG and its phase II metabolites in tissue micro-regions. Simultaneous visualization of EGCG and its phase II metabolites in (**A**–**E**) liver and (**F**–**J**) kidney sections. Images (**A**,**F**) show H & E staining; (**B**,**G**) show optical microscopy, and the other images are for MALDI-MS of EGCG (*m*/*z* 457) (**C**,**H**); EGCG-sulfate (*m*/*z* 537) (**D**,**I**), and EGCG-glucuronide (*m*/*z* 633) (**E**,**J**). An additional EGCG spot (red circle) was visualized as a positive, internal control. Adapted with permission from reference [5].

Understanding the metabolic fates of bioactive dietary polyphenols is indispensable for determining their in vivo molecular mechanisms [66]. Some studies have reported that green tea polyphenols are subjected to phase II biotransformation and predominantly undergo methylation, glucuronidation, and sulfation in the intestine, liver, and kidneys [67]. However, both the functions of the metabolites and their localizations in different tissue micro-regions remain unclear [68]. By contrast, 1,5-DAN-MALDI-MSI was able to visualize a spatially-resolved biotransformation based on simultaneous mapping of orally-dosed EGCG and its phase II metabolites, including its monosulfate (*m*/*z* 537) and monoglucuronide (*m*/*z* 633) forms (Figure 2) [5]. Interestingly, unlike liver, the localization patterns in the kidney compartments (pelvis, medulla, and cortex) were clearly different among EGCG and its phase II metabolites. In the kidney tissue extract, EGCG and its major conjugates (methylated, sulfated, and glucuronidated forms) were observed. The peak abundances of these three conjugates were markedly lower than that of EGCG. Nevertheless, both sulfated and glucuronidated forms were detected in MALDI-MSI measurements, but there was no peak for the methylated form. In negative ionization mode MALDI-MS, the phosphorylated compounds and carboxylic acids were efficiently ionized, indicating that compounds with leaving groups, including phosphate and carboxylic groups, readily undergo deprotonation [47,69,70,71]. Unlike methylation, sulfation or glucuronidation can introduce a leaving group (sulfate or carboxylic group, respectively) into EGCG. In MALDI-MS using 1,5-DAN, the introduction of such an ionizable leaving group may promote ionization and contribute to higher MALDI efficiency for EGCG phase II conjugates despite their low tissue abundances compared with EGCG [5]. Although the bioavailability of EGCG is very low [66,67], this sensing technology was able to visualize, for the first time, the in situ distribution of EGCG phase II metabolites in liver and kidney sections after oral dosing. The use of 1,5-DAN-MALDI-MSI will open new avenues for investigating the in situ metabolism of bioactive dietary polyphenols subjected to phase II biotransformation, and may help to accelerate the highly effective and efficient design of plant-derived pharmaceuticals, multicomponent botanical drugs, dietary supplements, and functional foods. The advantages of this MALDI-MS methodology include label-free imaging and simultaneous detection of an orally-dosed dietary polyphenol and its metabolites. This technique should help to overcome the drawbacks associated with conventional molecular imaging.

However, this technique could not be used to visualize EGCG and its phase II metabolites in kidney or liver tissue section after oral dosing at a normal intake level (20 mg/kg body weight) [5]. To overcome the limitation of the 1,5-DAN-MALDI-MSI technique and to ensure its practical use, further improvement of its detection sensitivity is required. This could be achieved by a targeted selective reaction monitoring mode approach using a triple quadrupole system [72], application of a Fourier transform-ion cyclotron resonance-MS (FT-ICR-MS) instrument capable of continuously accumulating selected ions [73], and on-tissue chemical derivatization approaches to increase the ionization efficiency [74,75]. In addition, the MALDI efficiency could be improved through matrix selection and development. Kim et al. reported that orally administered strictinin, an ellagitannin found in green tea, was detected as the intact form (*m*/*z* 633 [M − H]^−^) in mouse kidneys [76]. 1,5-DAN-MALDI-MSI, using the same high-vacuum pressure MALDI-TOF-MS instrument as in EGCG, detected an ion peak for this compound at *m*/*z* 633, but a clear ion image was not obtained. By contrast, an atmospheric pressure MALDI-quadrupole ion trap-TOF-MS instrument, called an iMScope, allowed clear visualization of strictinin, suggesting that selection of an appropriate MS instrument is important for sensitive detection of green tea polyphenols. Several studies have revealed that strictinin possesses various pharmacological properties, such as anti-oxidative, anti-viral, anti-diabetic, anti-allergic, and phagocytic activities [77,78,79,80,81]. It is known that ellagitannins are quite stable in the stomach and undergo a massive metabolic transformation in the gut to ellagic acid and urolithins, and these gut metabolites play an important role in the biological activities of ellagitannins [82,83]. In vivo bioactivity of an orally dosed strictinin has been reported [5], but there was no information on the amount of the intact strictinin within tissues and plasma. By contrast, Kim et al. showed, for the first time, the existence of intact strictinin in the kidneys 1 h after oral dosing. Visualization of the intact form in tissues other than the gastrointestinal tract may contribute to elucidation of the in vivo mechanism for the action of strictinin. In the future, other approaches including kinetic histochemistry [25,58,84], microscopic analysis with high spatial resolution [26,85,86], three-dimensional imaging [87,88,89], and analysis of the distribution of other metabolites [67,89] will be helpful to unravel both the biological consequences of biotransformation of dietary polyphenols and their mechanism(s) of action.

## 3. Evaluation of Green Tea Functionality Based on Its Compositional Balance

### 3.1. Metabolic Profiling

**Figure 3:**
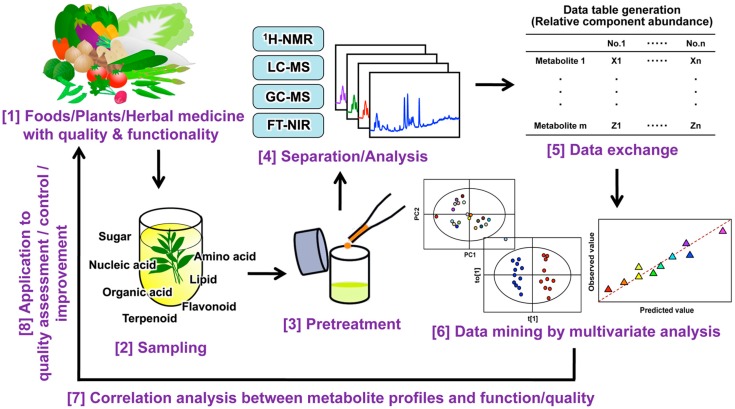
Process flow for a typical metabolic profiling experiment.

**Figure 4:**
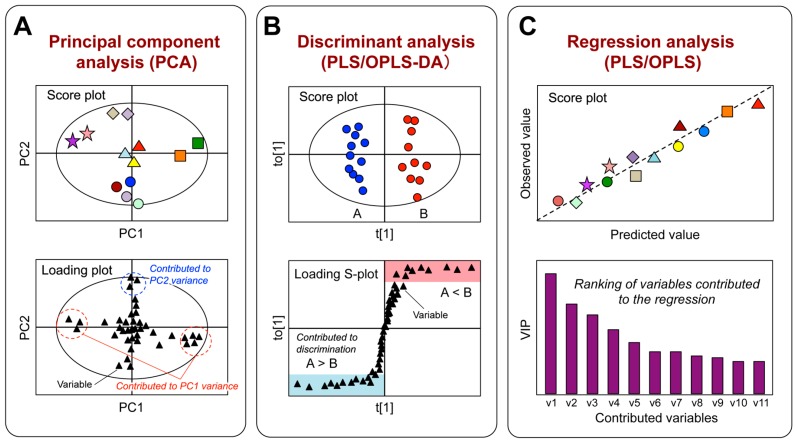
Multivariate statistical analyses used in metabolic profiling. (**A**) PCA provides an overview of all samples (color symbols) in a dataset. The score plot can visualize the relationships among samples on a two-dimensional model plane (PC1 and PC2). The loading plot can show variables contributed to score formation. Selected variables (dotted circles) contribute to the separation of samples along the PC axes; (**B**) The supervised method, PLS-DA or OPLS-DA, is used to isolate the variables responsible for discriminating the difference between samples (**A**,**B**). The loading S-plot, a plot of the covariance versus the correlation in conjunction with the variable trend plots, allows easer visualization of the data. The variables that changed most significantly are plotted at the top (red zone) or bottom (blue zone) of the S-shape plot, and those that do not vary significantly are plotted in the middle; (**C**) PLS or OPLS regression analysis is chemometric projection method relating two independent variables (X and Y) via a liner multivariate model. This method can extract Y-correlated information from the X-variable data. Bar chart shows influence of variables used to create Y predictor for samples.

The chemical components of tea vary according to the species/cultivar, environment, growth, storage conditions, and leaf quality [90]. In most cases, the quality of a tea and its bioactive functions (i.e., the health-promoting effects in human and animal models) are defined by its specific composition. The functional biochemistry of plants is very diverse, the concentrations of many compounds vary widely, and metabolomic analyses are required to simultaneously determine a broad range of metabolites in plant extracts. Among the many analytical platforms, MS is the most sensitive and selective technique, and thus it is the method of choice for metabolomic research on plants [91]. Liquid chromatography (LC)-MS and gas chromatography (GC)-MS are used extensively to investigate a wide range of molecules, including primary and secondary metabolites [92]. The workflow of metabolic profiling (MP) includes sample preparation, analysis using various instruments, data processing, and data analysis (Figure 3). It is crucial that data analysis method can detect significant changes so that the data from biological samples can be validated. Generally, multivariate statistical analysis is used to clarify similarities and differences among samples based on the multivariate data matrix (e.g., MS datasets). Such relationships are usually displayed as scatter plots (score plots) (Figure 4A). Because hundreds of variables (peaks) are obtained, the relationships among samples must be theoretically interpreted on hundreds of dimensional axes (variables), but these relationships cannot be simply displayed. To visualize the sample characteristics, multivariate analysis can extract sample features by dimensional reduction. That is, hundreds of original variables are decreased to two or three synthetic variables, which are orthogonal with each other. This maximizes the statistical variance of the samples, while leaving the original features of the samples largely unaffected [93]. The synthetic variables consist of hundreds of original variables. An understanding of the contribution of each original variable to the synthetic variables leads to the identification of key variables that contribute to the similarities or differences among the samples (Figure 4A, loading plot).

Principal component analysis (PCA) is an unsupervised approach and the most frequently used method in metabolomics for data mining (Figure 4A). The results are depicted as a score plot and include two synthetic variables: principal component (PC) 1 (with the greatest variance) and PC2 (with the next greatest variance, orthogonal with PC1). This displays intrinsic groups of samples based on spectral variations. The corresponding loading plots show the contribution of each spectral variable to score formation. The supervised multivariate technique is also used for identifying interesting metabolites. The partial least-squares (PLS)-based approach, which includes PLS, PLS-discriminant analysis (DA), orthogonal PLS (OPLS), and OPLS-DA, can extract Y-correlated information from the X matrix (Figure 4B,C). The X matrix is an organized data matrix obtained with the non-targeted approach, while the Y matrix is the supervised data. If the Y matrix has specific variables, such as the parameter variables of a producing instrument or food quality evaluated by a sensory test, PLS and OPLS approaches are convenient methods for extracting meaningful metabolites correlated with Y variables. Particularly in the univariate Y, an OPLS approach allows easy interpretation of the results compared with PLS. The orthogonal approach can remove uncorrelated data from the X to Y matrix, where the number of latent variables correlated to Y is generally one. Because of the features of the orthogonal method, users only focus on the first component to interpret the results. When searching for significant metabolites, both the loading matrix and the variable importance in the projection (VIP) can be used (Figure 4C). If the Y matrix does not have specific variables, but has some biases, a discriminant approach, such as PLS-DA or OPLS-DA, is frequently used (Figure 4B).

Metabolomic studies coupled with chemometric methods including PCA and PLS regression analysis have been used to explore the relationships between the metabolome of diverse plant species and their genotypes, origins, vintages, qualities, and other specific attributes [94,95,96,97,98,99]. MP techniques are often used to evaluate the nutraceutical (nutritional or physiological) value of a single plant cultivar for quality control and breeding. In the field of nutraceutical (functional food) research, such techniques have been used to identify subtle metabolic differences among individuals or different environmental conditions (e.g., diet) [100]. This chemometric approach can clarify similarities or differences among samples by compositional balance based on the relative abundance of each metabolite to the total abundance of all metabolites. Additionally, such a technique enables us to theoretically calculate the relative contribution of all multicomponent factors detected in crude samples to the total functionality. Considering the principle of this methodology, it is expected that MP may become an effective strategy for obtaining a comprehensive understanding of the attributes of crude samples, including multicomponent foods (agricultural products) and medicinal herbs. In the functional food research field, various functionalities are evaluated by activity and abundance of a single particular component in foods, but simultaneous evaluation of coexisting multicomponent factors is required for elucidating real functionality in multicomponent systems. However, to date, there has been little research on the use of MP to compare or predict the nutraceutical (bioactive) properties (i.e., health-promoting effects in human and animal models) of many plant cultivars. Therefore, elucidating the relationship between the metabolome and the bioactivities of diverse cultivars could be a novel strategy for identifying the nutraceutical potentials of various plant cultivars for functional food and botanical drug designs.

### 3.2. Quality Evaluation Based on Chemical Composition

Based on the aforementioned analytical strategies, MP of extracts from tea leaves has been performed to study various attributes (Table 2). Generally, the quality of a tea is evaluated by professional tea tasters who evaluate product quality using the leaves’ appearance and aroma, color, and taste of the brew.

Because the process of training a skilled tea taster may take years and is very expensive, instrumental techniques for evaluating tea quality are attractive. As a promising alternative approach to the traditional methods of chemical and sensory analysis, MP has been evaluated for tea analysis. Fukusaki and coworkers succeeded in predicting the ranking of Japanese green tea samples by MP using four different analytical platforms [101,102,103,104]. Furthermore, metabolites that played an important role in grade classification of green tea were identified. These chemometric or data-driven approaches might be advantageous over the conventional sensory test for classification and determination of tea quality. Recently, metabolites from a 50% aqueous methanol extract of green teas grown with different shade periods (0, 15, 18, and 20 days) were analyzed to investigate the effect of low light on their nutritional and sensory qualities [105]. The shaded groups could be clearly distinguished from the control (0 day), and the 20-day group could be separated from the 15- and 18-day groups. The shade treatment increased the levels of quercetin-galactosylrutinoside, kaempferol-glucosylrutinoside, epicatechin-3-*O*-gallate, EGCG, tryptophan, phenylalanine, theanine, glutamine, glutamate, and caffeine but decreased those of quercetin-glucosylrutinoside, kaempferol-glucoside, gallocatechin, and epigallocatechin. This result, along with the sensory evaluation and color data, suggested that shade improved the nutritional and sensory qualities of green tea. They also proposed a metabolomic pathway that could explain the relationship between low light and tea quality.

### 3.3. Analysis of Metabolic Responses to Tea Consumption

Metabolomics approaches have been reported as important and effective tools to examine changes in endogenous metabolites in the whole system and potentially provide a better mechanistic understanding of biochemical and cellular events [118]. Recently, the effects of black and green tea consumption on the human metabolism were investigated [119]. Green and black tea consumption (equivalent to 12 cups of tea per day) had different impacts on endogenous metabolites in urine and plasma. Green tea intake increased the urinary excretion of several citric acid cycle intermediates more than black tea, which suggests green tea flavanols affect the human oxidative energy metabolism and/or biosynthetic pathways. Hodgson and coworkers showed that GC-MS and LC-MS-based MP of human plasma could enhance our understanding of the mode of action of exercise and GTE beyond the physiological outcomes [120]. Moderate exercise stimulated multiple metabolic pathways including lipolysis, glycolysis, the citric acid cycle, and the adrenergic system. Metabolite changes induced by GTE were subtler and affected fewer pathways when compared with those induced by exercise alone. Supplementation with GTE for 7 days (1200 mg total catechins and 240 mg caffeine/day) mainly enhanced metabolites indicative of lipolysis and fat oxidation under resting conditions when compared with a placebo. This effect was not enhanced during exercise. Furthermore, GTE did not stimulate the adrenergic system during rest and exercise because no increase in noradrenaline and related catecholamines was observed. This result challenges the idea that catechol-*O*-methyltransferase inhibition as the putative mechanism of action of GTE in vivo. Yet GTE stimulated lipolysis under resting conditions, which suggested nonadrenergic mechanisms were involved.

Green tea is thought to have beneficial health effects, including protective effects against oxidative stress. Acetaminophen (APAP) is a widely used analgesic drug that can cause acute liver injury in overdose situations. Lu et al. explored the effects of GTEs (500 or 1000 mg/kg) on APAP-induced hepatotoxicity in liver tissue extracts using MP of mice livers after GTE pretreatment 3 h or 3 days before APAP exposure or GTE exposure 6 h after APAP [121]. GTE given before APAP ameliorated the APAP-induced hepatotoxicity in a dose-dependent manner, whereas GTE given after APAP potentiated the toxicity. APAP exposure alone significantly altered multiple metabolite levels compared with the control. By comparison, with GTE pretreatment, the metabolite levels returned to control levels or showed lower changes. By contrast, GTE given after APAP caused greater changes in the metabolite levels than APAP alone, indicating more severe hepatotoxicity. The changes in liver metabolites indicated perturbations of fatty acid, energy, bile acid, and phospholipid metabolisms induced by APAP, with differing effects on these metabolites depending upon the time of GTE exposure. The results indicated that the time at which GTE was given greatly influenced the severity of APAP-induced toxicity. These findings highlight the need to understand the interactions between GTE and drugs, and support the importance of a metabolomic approach. 

### 3.4. Evaluation of Health-Promoting Effects

**Figure 5:**
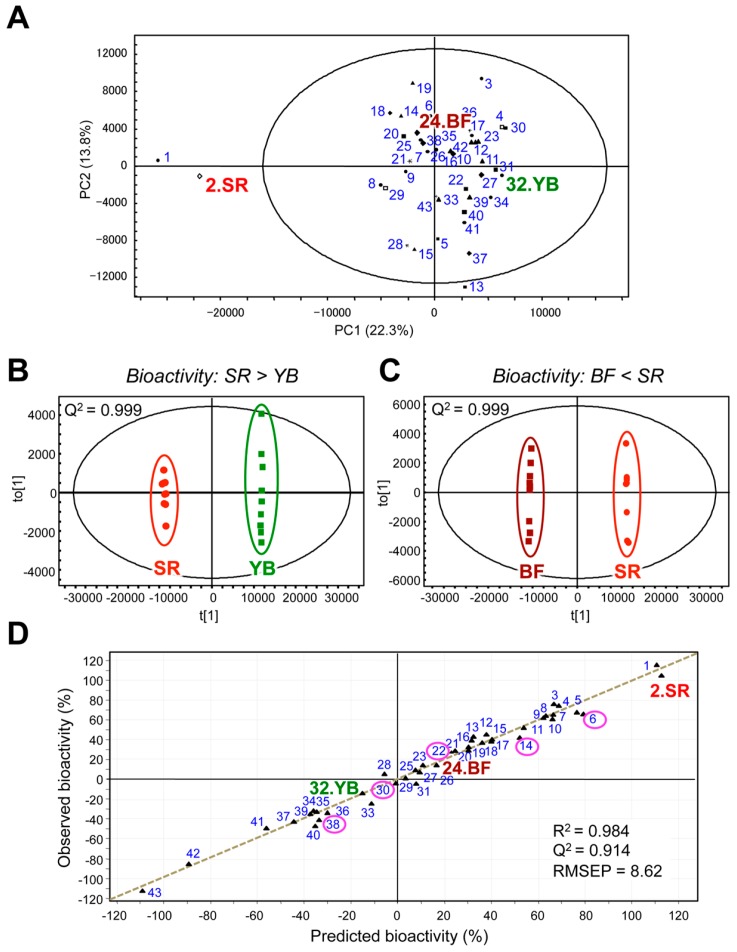
Multivariate statistical analyses of metabolic profiles of diverse green tea cultivars and their bioactivities. (**A**) PCA score plot shows separate clustering of metabolic profiles corresponding to Nou-6 and SR, and other cultivars. The number in the figure indicates the bioactivity ranking of the tea cultivar; OPLS-DA score plots (**B**,**C**) discriminate between the more bioactive SR cultivar and other cultivars, such as the non-bioactive Yabukita (YB) cultivar and the less bioactive Benifuuki (BF) cultivar. The predictive ability parameter Q^2^ was 0.999, indicating that the OPLS-DA models were reliable; (**D**) The bioactivity-prediction PLS regression model was calculated from 43 tea samples included in 38 training and 5 test (pink circle) sets. The correlation coefficient R^2^ and the cross-validated correlation coefficient Q^2^ were both more than 0.9. The validation error, value root mean squared error of prediction (RMSEP), was less than 5% (8.62 = 3.8%). These results indicated that the PLS regression model was reliable. Adapted with permission from reference [8].

GTEs have various health benefits, and these vary from cultivar to cultivar. Although there are numerous tea cultivars, little is known about the differences in their nutraceutical properties. We performed metabolomic analyses to explore the relationship between the metabolome and health-promoting attributes of a diverse range of Japanese green tea cultivars [8]. We investigated the abilities of leaf extracts from 43 Japanese green tea cultivars to inhibit thrombin-induced phosphorylation of myosin regulatory light chain (MRLC) in human umbilical vein endothelial cells. This thrombin-induced phosphorylation is a potential hallmark of vascular endothelial dysfunction. Among the tested cultivars, Cha Chuukanbohon Nou-6 and Sunrouge (SR) strongly inhibited MRLC phosphorylation. To evaluate the bioactivities of green tea cultivars using a metabolomics approach, metabolite profiles of all tea extracts were determined by LC-MS (Figure 5). Multivariate analyses revealed differences among the green tea cultivars with respect to their abilities to inhibit MRLC phosphorylation. In the SR cultivar, polyphenols were associated with its unique metabolic profile and its bioactivity. In addition, using PLS regression analysis, we constructed a reliable bioactivity-prediction model to predict the inhibitory effect of a tea cultivar based on its metabolome.

**Figure 6:**
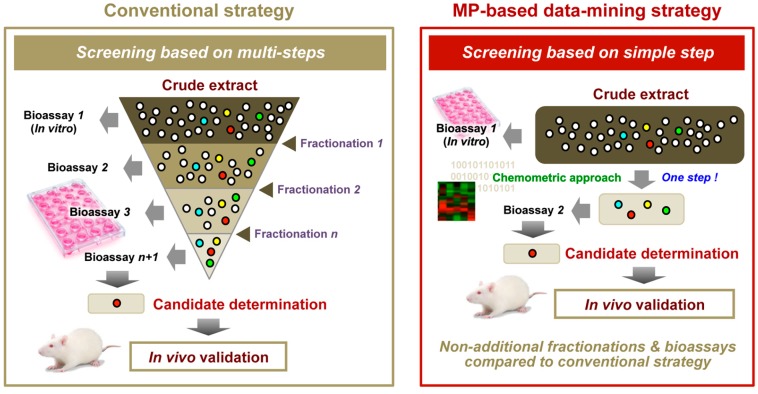
MP-based data-mining for an effective chemical combination. Adapted with permission from reference [116].

This model was based on certain identified metabolites that were associated with bioactivity. Intriguingly, when added to an extract from the non-bioactive cultivar Yabukita (YB), several metabolites enriched in SR were able to transform it into a bioactive extract [8]. Generally, most bioactive phytochemicals are isolated from crude extracts by fractionation and purification, during which low-abundance but potentially interesting compounds may be lost. By contrast, the aforementioned chemometric approach allows for the identification of the promising compounds in a crude mixture containing many compounds at different concentrations in a single analysis without fractionation or purification steps prior to LC-MS as well as additional bioactivity assessment after LC-MS. This strategy may prove valuable for the isolation of additional bioactive compounds from GTEs (Figure 6).

Recently, Ku et al. used metabolomic approaches to determine the effect of manufacturing type or cultivation method on the chemical compositions of a single tea cultivar (green tea or pu-erh tea) [111,114]. They suggested that several polyphenolic compounds were associated with manufacturing type, cultivation method, or anti-oxidant activity. In our research, we investigated relationships between metabolomic data and the health-promoting effects in 43 green tea cultivars. In the SR cultivar, we found certain polyphenolic constituents (delphinidin-glucoside/galactoside, quercetin-glucoside/galactoside, and theogallin) were associated with bioactivity [8]. These polyphenols differed from those reported by Ku et al. [111,114]. In addition, the levels of polyphenols, especially anthocyanins, were very high in the SR cultivar, but very low in the most consumed and distributed Japanese green tea cultivar YB. Although polyphenols have many health benefits, the relationships between these compounds and the inhibition of MRLC phosphorylation in human endothelial cells remains unclear. These facts indicate that a metabolomic approach is useful for identifying unique bioactive factors. Such information may be useful for the development of markers to produce new cultivars with greater bioactivity, and to screen for bioactive tea cultivars. 

A MP approach enables us to obtain compositional information based on a correlation between relative component abundance and bioactivity (functionality), which is indispensable for a better understanding of green tea functionality under multicomponent systems. This chemometric methodology can easily calculate the relative functionality of all coexisting multicomponent factors detected in GTEs. This information may contribute new scientific evidence that could be useful for the development of green tea-based functional foods, multicomponent botanical drugs, and preferable combinations of foods/beverages for health benefits.

### 3.5. Selection of Bioactivity-Related Chemical Combination

It is known that GTE induces apoptosis of cancer cells without adversely affecting normal cells. In several clinical trials, GTE was well tolerated and had potential anti-cancer activity [122,123,124,125,126]. In the phytochemical libraries of GTE, EGCG is the primary compound responsible for the anti-cancer effect of GTE, but the effect of EGCG alone is limited. The potential of other compounds to act synergistically with EGCG in GTE has not been examined. To identify GTE compounds capable of potentiating EGCG bioactivity, Kumazoe et al. performed LC-MS-based MP of multiple GTE panels, and created an OPLS regression model using GTE composition profiles and bioactivity for induction of apoptosis using multiple myeloma [116]. The bioactivities of the GTEs were explained by their composition profiles. In this model, compounds highly relevant for explaining predicted apoptosis-inducing effects were also identified from VIP values. Large VIP values (>1) are the most relevant for explaining the predicted bioactivity. To screen candidates for effective anti-apoptotic combinations, compounds with high VIP rankings were selected. Among these compounds, only the polyphenol eriodictyol significantly potentiated the anti-cancer effect of EGCG in vitro and in a mouse tumor model by amplifying EGCG-induced activation of the 67-kDa laminin receptor (the cell-surface EGCG-sensing molecule)/protein kinase B/endothelial nitric oxide synthase/protein kinase C delta/acid sphingomyelinase signaling pathway. These results suggest that MP is an effective chemical-mining approach for identifying botanical drugs with therapeutic potential against multiple myeloma (Figure 6). Furthermore, these findings highlight the potential application of MP techniques to evaluate the pharmacological effects of diverse compounds in raw plant extracts and to screen for anti-cancer compounds or synergetic sensitizers. This metabolomic screening approach with supervised multivariate OPLS regression analysis could be a valuable strategy for preclinical identification of anti-cancer compounds.

Although understanding their chemical composition is vital for accurately predicting the bioactivity of multicomponent drugs, nutraceuticals, and foods, no analytical approach exists to easily predict the bioactivity of multicomponent systems from the complex behaviors of multiple coexisting factors. By contrast, MP enables theoretical calculation of the relative contributions of all multicomponent factors detected in crude samples to the total bioactivity. Considering the principle of this methodology, it is expected that MP may become an effective strategy for obtaining a comprehensive understanding of the physiological activities of multicomponent drugs and nutraceuticals. Recently, we demonstrated that the MALDI-MS-MP technique could be used to evaluate the anti-oxidant activity, oxygen radical absorbance capacity (ORAC), of diverse GTE panels based on their compositional balances, and select an effective chemical combination to predict the bioactivity [117]. 

**Figure 7:**
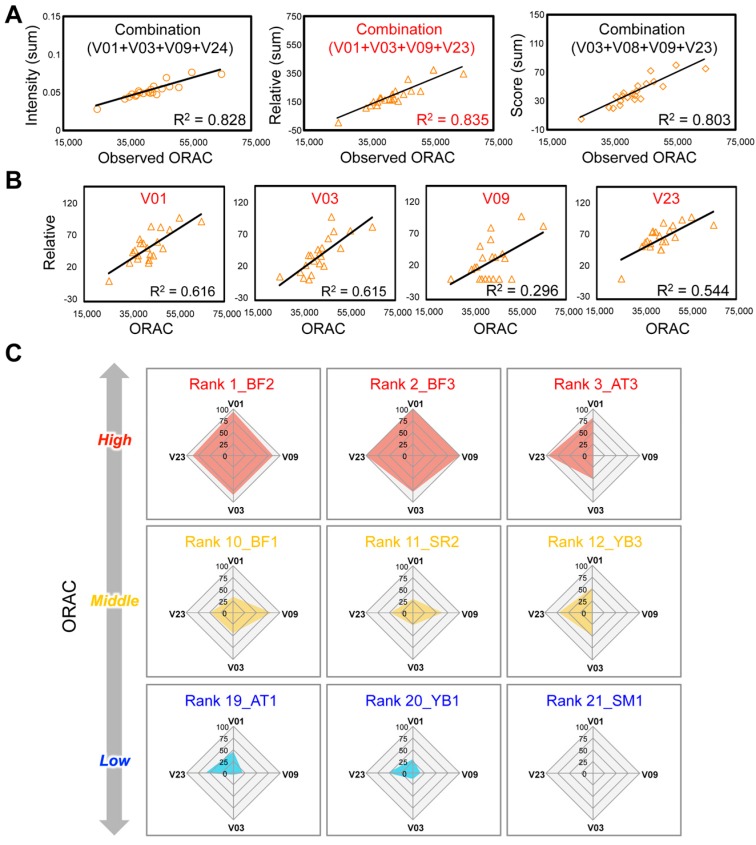
Chemometrics-driven selection of bioactivity-correlated chemical combinations in GTEs and visualization of observed ORAC values using the selected combination. (**A**) The highest correlation was found between the observed ORAC value and the summed abundance (Intensity) of four components as a bioactivity-predictive combination. Correlations based on the relative value (Relative: Maximum, 100; Minimum, 1) and ranked scored value (Score: Top, 21; Bottom, 1) of the summed abundances are also shown; (**B**) Correlation of the Relative value of each individual component with the ORAC; (**C**) Observed ORAC values of GTEs visualized as radar charts using information from the four selected components. Selected representative charts of the GTEs are shown, demonstrating that the ORAC can be visually estimated from the abundances of the four components. Adapted with permission from reference [117].

The chemometric procedure OPLS regression analysis allowed the evaluation of GTE bioactivity from multicomponent rather than single-component information (Figure 7A,B). The bioactivity could be easily evaluated by calculating the summed abundance of a few selected components that contributed most to construction of the prediction model. Furthermore, the chart visualization was an effective strategy to easily understand ORAC values using the selected component combination (Figure 7C). This finding suggests a promising strategy for efficiently selecting candidate combinations from multivariate data of multiple sample panels with diverse bioactivities, which is important but technically challenging in pharmaceutical, nutraceutical, and functional food research, where single-sample panels are dominant. In conventional research on the evaluation of quality and bioactivity, the goal is usually to isolate a single component from crude mixtures and use its abundance to predict the sample’s properties. By contrast, the aforementioned study has shown that bioactivity can be predicted using multicomponent information (i.e., the abundances of a combination of components), the accuracy of which depends on the chosen combination of components and the choice between three abundance measurements (Intensity, Relative, or Score) (Figure 7A). These efforts will enhance understanding of chemometrics procedures, and contribute to development of an effective and simple means of data presentation using multicomponent information. This information may benefit the application of multivariate statistical methodology to the evaluation of the bioactivities in crude multicomponent systems. 

In pharmaceutical, nutraceutical, and food functionality research, the conventional evaluation method for bioactivity, targeted analysis, attempts to predict the total bioactivity of an entire sample by measuring the activity and abundance of a single component. However, this approach carries a risk of overestimating or underestimating bioactivity by neglecting the potential interfering effects of multiple coexisting factors. In addition, such an approach cannot easily calculate the relative contributions of all coexisting factors to the total bioactivity of an entire sample. Furthermore, the screening of bioactivity-related chemical combinations from crude samples is generally time-consuming, expensive, and labor-intensive because of the multiple, repetitive processes for fractionation and the bioassay (Figure 6). The results of the aforementioned study suggested that a chemometrics-based and non-targeted MP approach, using multiple GTE panels with diverse bioactivities, could overcome these drawbacks.

## 4. Conclusions

For specifically sensing complex behaviors of low-molecular-weight phytochemicals in the body or a tea leaf infusion, we have proposed new concepts and techniques. This includes an in situ label-free MALDI-MS imaging technique for visualizing spatially-resolved biotransformation based on simultaneous mapping of orally-dosed EGCG and its phase II metabolites, and a chemometrics approach capable of discriminating and predicting the functionalities of multicomponent GTEs from their compositional balances and calculating the relative functionalities of all components. These findings will provide important insight into functionality-related interactions between phytochemicals and the body. This approach should overcome the drawbacks (low specificity, time and labor requirements, expensive, and lack of ability to analyze multiple components) associated with conventional molecular-sensing techniques. Beneficial information on the usefulness of phytochemical-sensing technology in GTE research will emerge from further biological applications such as disease model analysis and human intervention trials. This review will be applicable to studies of a wide range of other foods and herbal medicines.

## Figures and Tables

**Table 1:** Application of laser-based MSI to analysis of phytochemicals in edible plants.

Matrix	Analyte	Tissue	Species	Ref.
DHB	γ-Oryzanol, α-tocopherol, phytic acid	Seed	Rice	[42]
	Anthocynanins, lipids	Seed	Black rice	[44]
	GABA, amino acids, sugars	Fruit	Eggplant	[41]
	Glycoalkaloids	Tubers	Potato	[53]
	Anthocyanins	Fruit	Blueberry	[43]
	Tomatine, esculeoside A	Fruit	Tomato	[49]
CHCA	Oligosaccharides	Stem	Wheat	[54]
	Amino acids, sugars, sugar phosphates	Grain	Wheat	[55]
	Capsaicin	Fruit	Capsium	[56]
	Ginsenosides	Root	Ginseng	[56]
	Flavonoids, dihydrochalcones	Fruit	Apple	[57]
9-AA	Amino acids, sugars, sugar phosphates	Grain	Wheat	[55]
	Caffeic acid, organic acids, amino acids, nucleotides	Fruit	Tomato	[49]
THAP	Flavonoids, organic acids	Fruit	Strawberry	[50]
Matrix-free	Stilbenoids	Leaf	Grapevine	[52]
	6-Gingerol, monoterpene	Rhizome	Ginger	[26]

**Table 2:** Metabolic profiling of tea extracts for quality evaluation.

Targets	Products (Number)	Ref.
Production origin/Price and grade	Green tea (191)	[90]
Production origin/Manufacturing type	Green, oolong, black, yellow, white, and pur-erh teas (187)	[106]
Climate	Green tea (4)	[107]
Altitude	Black tea (4)	[108]
Manufacturing type/Age	Pu-erh, black, and green teas (24)	[109]
	Tuocha (Black, green, or postfermented type; 20)	[110]
Shade culture/Season	Green tea (4)	[111]
Shade period/Nutritional and sensory qualities	Green tea (4)	[105]
Fermentation process	Pu-erh tea (7)	[112]
	Pu-erh, black, green, white, yellow, and oolong teas (71)	[113]
Postfermentation year	Pu-erh tea (30)	[114]
Plucking position of leaf	Green tea (5)	[115]
Sensory quality	Green tea (53)	[104]
	Green tea (56)	[103]
	Green tea (53)	[101]
	Green tea (64)	[102]
Health-promoting effect	Green tea (43)	[8]
	Green tea (43)	[116]
	Green tea (21)	[117]
